# Hydrocarbon Contamination Decreases Mating Success in a Marine Planktonic Copepod

**DOI:** 10.1371/journal.pone.0026283

**Published:** 2011-10-28

**Authors:** Laurent Seuront

**Affiliations:** 1 School of Biological Sciences, Flinders University, Adelaide, Australia; 2 Aquatic Sciences, South Australian Research and Development Institute, West Beach, Australia; 3 Centre National de la Recherche Scientifique, UMR LOG 8187, Wimereux, France; 4 Department of Physics, Center for Polymer Studies, Boston University, Boston, Massachusetts, United States of America; Institute of Marine Research, Norway

## Abstract

The mating behavior and the mating success of copepods rely on chemoreception to locate and track a sexual partner. However, the potential impact of the water-soluble fraction of hydrocarbons on these aspects of copepod reproduction has never been tested despite the widely acknowledged acute chemosensory abilities of copepods. I examined whether three concentrations of the water-soluble fraction of diesel oil (0.01%, 0.1% and 1%) impacts (i) the swimming behavior of both adult males and females of the widespread calanoid copepod *Temora longcornis*, and (ii) the ability of males to locate, track and mate with females. The three concentrations of the water-soluble fraction of diesel oil (WSF) significantly and non-significantly affect female and male swimming velocities, respectively. In contrast, both the complexity of male and female swimming paths significantly decreased with increasing WSF concentrations, hence suggesting a sex-specific sensitivity to WSF contaminated seawater. In addition, the three WSF concentrations impacted both *T. longicornis* mating behavior and mating success. Specifically, the ability of males to detect female pheromone trails, to accurately follow trails and to successfully track a female significantly decreased with increasing WSF concentrations. This led to a significant decrease in contact and capture rates from control to WSF contaminated seawater. These results indicate that hydrocarbon contamination of seawater decreases the ability of male copepods to detect and track a female, hence suggest an overall impact on population fitness and dynamics.

## Introduction

Coastal ecosystems are increasingly threatened by a variety of anthropogenic pollutants. Specifically, one of the most dramatic contaminations of coastal waters is related to accidental crude oil spills, such as *Torry Canyon* (1967), *Amoco Cadiz* (1978), *Exxon Valdez* (1989), *Aegean Sea* (1992), *North Cape* (1996), *Nakhodka* (1997), *Erika* (1999), *Prestige* (2002) and more recently the *Deepwater Horizon* wellhead in the Gulf of Mexico (2010), that severely impacted coastal marine communities. However, leaks from ships, petroleum transport, refining and intentional spills are important sources of polycyclic aromatic hydrocarbons (PAHs) in the ocean, particularly in coastal and shelf waters [1]. PAHs have become increasingly important because of their potential carcinogenicity, mutagenicity and teratogenicity to aquatic organisms and man [2]. In this context, understanding the direct and indirect links between hydrocarbon contaminants and marine species is critical, especially for minute planktonic organisms such as copepods that play a main role in the functioning of marine ecosystems and in biogeochemical cycles [3].

Oil slick episodes may induce mass mortalities in zooplankton in general and copepods in particular [4]–[6]. However, a more pernicious consequence of PAHs resides in the sub-lethal effects induced by their water-soluble fraction that affects copepod physiology, feeding and fecundity [7]–[9], hence abundance and diversity [10]. Despite the amount of work devoted to copepod chemoreception [11]–[14] and the evidence for copepods to modify their swimming behavior in response to naphthalene contamination [15] and to detect, avoid and escape localized patches contaminated by the soluble fraction of diesel oil [16], the potential for PAHs to disrupt copepod chemosensory abilities has yet to be investigated, although this would have considerable implication for mating success, hence population dynamics.

Finding mates is challenging in the ocean for copepods, as they typically rely on non-visual senses for detecting, identifying and locating mates in a three-dimensional environment, where concentrations of adults are as low as a few individuals per cubic meter [17]. Typically, males searches for chemical cues signalling the presence and position of the females. In some species, such as the widely distributed coastal calanoid copepod *Temora longicornis*, females leave a chemical trail in their wake, which males may sense and follow using chemical gradients along and across the trail to detect and locate the female [11]–[14]. Any disturbance of male chemosensory system may result in a reduced or loss of ability to detect females, which might lead to a decrease in mate encounter rates, hence mating rates.

Here, I evaluated whether the swimming behavior, and both the mating behavior and mating success of the calanoid copepod *Temora longicornis* are likely to be affected by hydrocarbon contamination of seawater. Specifically, the ability of males (i) to detect and follow the pheromone plume produced by conspecific females, and (ii) to capture them was evaluated in pure seawater and in seawater contaminated by the water-soluble fraction of diesel oil. A specific attention has been given to low concentrations of the water-soluble fraction of diesel oil (0.01%, 0.1% and 1%) that are well below the lethal concentration for *T. longicornis* to assess the impact of a chronic exposure to low concentrations of petroleum hydrocarbons.

## Methods

### Study species, sampling and acclimatization

*Temora longicornis* is very abundant in coastal temperate waters of the Northern Hemisphere [18]. It represents from 35 to 70% of the total population of copepods in the Southern Bight of the North Sea [19], [20] and is able to remove up to 49% of the daily primary production [21]. Its naupliar stages significantly contribute to larval fish diet [22]. *T. longicornis* were collected with a WP2 (200-µm mesh size) from the inshore surface waters of the Eastern English Channel (50°40′75″N, 1°31′1″E) at a temperature of 18°C and a salinity of 32 PSU. Specimens were subsequently gently diluted in 30-liter isotherm tanks using *in situ* seawater and transported to the laboratory where adult males and females were immediately sorted by pipette under a dissecting microscope. *T. longicornis* adults were then reared in 20-liter aquaria filled with filtered (Whatman GF/C glass-fibre filters, porosity 0.45 µm) seawater to which was added a suspension of the diatom *Skeletonema coastatum* at a concentration of 10^8^ cells l^−1^ and a mixture of *Isochrisis galbana* and *Nannochloropsis oculata* (3/4∶1/4) at a concentration of 10^7^ cell l^−1^. The larger heterotrophic flagellate *Oxyrrhis marina* was present as an additional food source at a concentration of 10^6^ cell l^−1^. *T. longicornis* were reared under constant conditions of temperature (18°C) and salinity (32 PSU) under a 12/12 light/dark cycle.

### Preparation of the soluble-fraction of diesel oil and preliminary toxicity assessment

**Figure 1:**
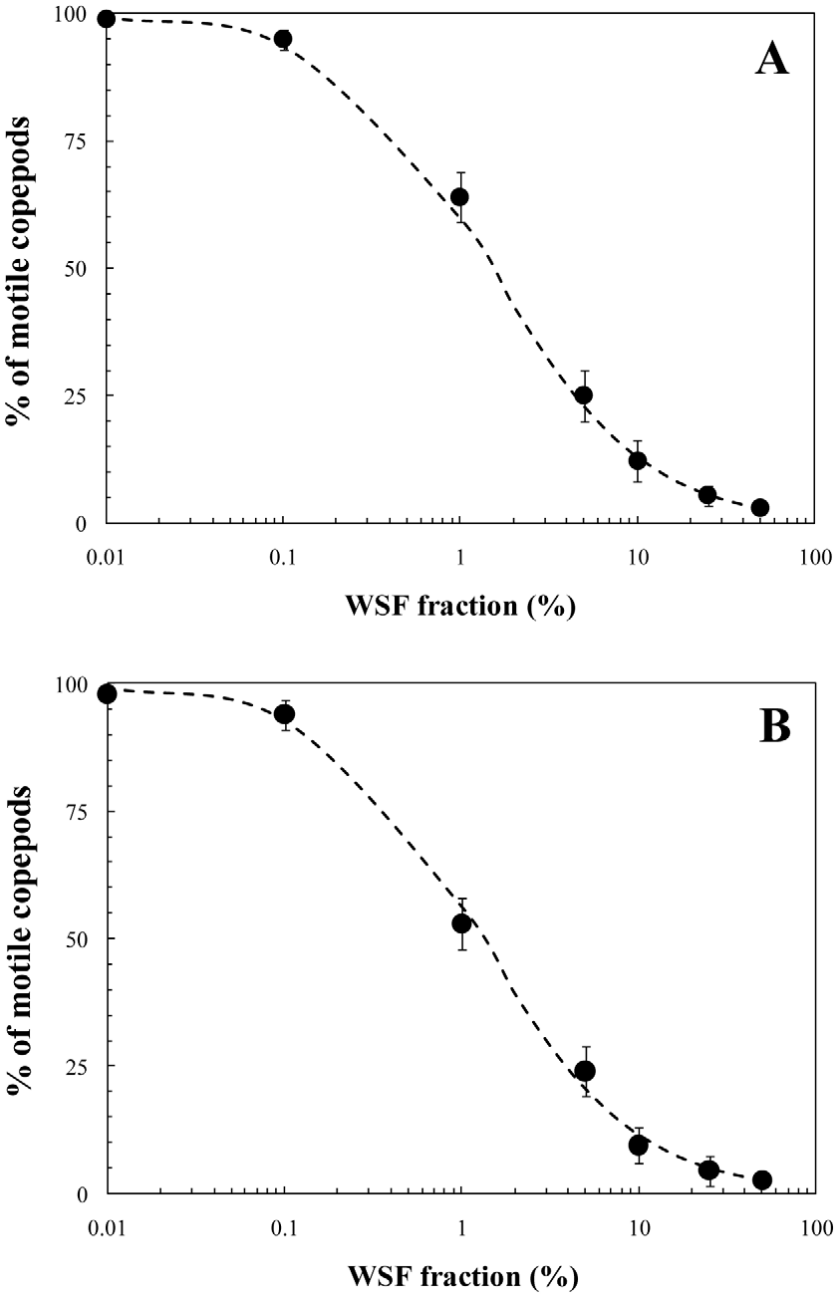
Survival response of adult *Temora longicornis* females (black circles) to a range of WSF concentrations (50%, 25%, 10%, 5%, 1%, 0.1% and 0.01%) over 24-h (A) and 48-h (B) toxicity assays. The data have been fitted to an allosteric decay model, see Eq. (1), with *r* ^2^ = 0.99 (A) and *r* ^2^ = 0.98 (B). Error bars are standard deviations.

The product considered as a potential contaminant of coastal waters was commercial diesel fuel oil. The water-soluble fraction of commercial diesel oil (WSF) was prepared stirring 1.8 l of filtered *in situ* seawater (Whatman GF/C filters) with 0.2 l of commercial diesel fuel oil for 2 h at 100 g. The mixed solution was allowed to stand for 24 h without stirring to separate the oil layer from the oil-saturated water. WSF stock solutions were siphoned into autoclaved, acid-rinsed glass containers and diluted with uncontaminated seawater at ‘high’ (1%), ‘medium’ (0.1%) and ‘low’ (0.01%) concentrations. The water-soluble fraction of oil and their derivatives products contain a mixture of polycyclic aromatic hydrocarbons (PAH), monoaromatic hydrocarbons often referred to as BTEX (benzene, toluene, ethylbenzene and xylenes), phenols and heterocyclic compounds, containing nitrogen and sulphur [23]–[25]. While technical limitations hampered the assessment of the precise chemical nature of the WSF stock solutions, the range of WSF concentrations has specifically been chosen to investigate the sub-lethal effects related to ‘natural’ background concentrations of pollutants [26], [27]. More specifically, among those compounds BTEX are the main class of hydrocarbons found in WSF [23], [28], and naphthalene is one of the most abundant polycyclic aromatic hydrocarbons dissolved in oil contaminated waters [29], [30] and has been widely used in toxicological assays [29], [31], [32]. BTEX and naphthalene concentrations are respectively in the range 450–35000 µg l^−1^ and 30–26000 µg l^−1^ in 100% water-soluble fraction [23], [25]. The ‘high’ (1%), ‘medium’ (0.1%) and ‘low’ (0.01%) concentrations used in the present work hence correspond to concentrations in the range 4.5–350 µg l^−1^, 0.45–35 µg l^−1^ and 0.045–3.5 µg l^−1^ for BTEX, and 0.3–260 µg l^−1^, 0.03–26 µg l^−1^ and 0.003–2.6 µg l^−1^ for naphthalene. These concentrations are all well below the lethal concentrations observed for a range of copepod species [7]–[9], [15], and for *Temora longicornis* over 24-h and 48-h toxicity assays. Briefly, the acute responses (mortality and narcosis) produced by the water-soluble fraction of commercial diesel oil followed a nonlinear allosteric decay model (*r* ^2^ = 0.99, *P*<0.01) defined as [8]:
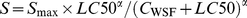
(1)where *S* and *S*_max_ are the survival rate (%) and the maximum survival rate (%), 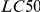 the concentration at which 50% of the specimens died, *C*_WSF_ the experimental WSF concentration (50%, 25%, 10%, 5%, 1%, 0.1% and 0.01%) and 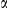 a fitting parameter. 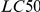 after 24-h and 48-h exposures were 7.5% and 12.7%, respectively (Fig. 1). Note that to discriminate mortality from narcotization, after a period of 4-h in clean seawater animals were examined again to assess the degree of recovery [31]. No narcotic effects were observed and the mortality and narcosis were hence very similar, i.e. 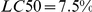 and 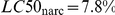, and 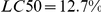 and 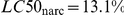 for the 24-h and 48-h toxicity assays, respectively. No significant differences were found in 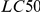 and 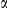 between males and females (*P*>0.05). The behavioral experiments were conducted with the same WSF stock solutions than the above-mentioned toxicity assays for mortality and narcosis.

### Behavioral experiments

Contaminated and uncontaminated behavioral experiments were replicated five times; each experiment involved three adult males and three adult females. WSF stock and working solutions were prepared 24 h before the behavioral experiments took place. The control experiments and the treatments were randomized, as well as the replicates of each experimental condition. The experimental chamber was rinsed with acetone, GF/C filtered seawater and distilled water, and allowed to dry between trials to remove any chemical compound. Prior to each experiment, the six experimental individuals were transferred in a filming set-up (a 15 cm×15 cm×15 cm glass chamber) filled up with uncontaminated seawater and the corresponding treatments, and were allowed to acclimatize for 15 min [16], [33]. All experimental individuals were used only once, and no narcosis or mortality was ever observed on any of the 120 tested individuals. Three-dimensional trajectories of *T. longicornis* males and females were recorded at a rate of 25 frame s^−1^ using two orthogonally oriented and synchronized infrared digital cameras (DV Sony DCR-PC120E) facing the experimental chamber. Six arrays of 72 infrared-light-emitting diodes (LEDs), each mounted on a printed circuit board about the size of a business card (i.e. 9.3 cm long and 4.9 cm wide) connected to a 12-volt DC power supply, provided the only light source from the bottom of the chamber. The cameras overlooked the experimental chamber from the side, and the various components of the set-up were adjusted so that the copepods were adequately resolved and in focus. Each experiment lasted 60 min, after which valid video clips were selected for analysis. Valid video clips consisted of pathways in which the animals were swimming freely, at least two body lengths away from any chamber's walls or the surface of the water [16], [33]. All the experiments were conducted at 18°C and 32 PSU in the dark and at night to avoid any potential behavioral artifact related to the diel cycle of the copepods [34]. Selected video clips were captured (DVgate Plus) as MPEG movies and converted into QuickTime TM movies (QuickTime Pro), after which the 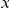, 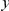 and 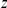 coordinates of swimming pathways were automatically extracted and subsequently combined into a 3D picture using LabTrack software (DiMedia, Kvistgård, Denmark). The time step was always 0.04 s, and output sequences of 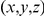 coordinates were subsequently used to characterize the motion behavior.

### Behavioral analysis

*Temora longicornis* swimming and mate-searching behaviors have been well described [11], [13], [16], [34]–[40]. Typically, females leave behind them a pheromone trail whose shape and dimensions depend on swimming behavior. Once males have detected the trail, they use chemical gradients along and across the pheromone trail to detect and locate the female. The swimming behavior of *T. longicornis* was hence classified as non-mating and mating behavioral sequences based on male swimming behavior. Non-mating sequences were obtained from male swimming paths occurring before the initiation of mate-searching behavior, i.e. when a male swam independently of female trails; mating sequences were obtained from male swimming paths occurring after the initiation of mate-searching behavior. Mating behavioral sequences were categorized as (i) tracking events: the male detects the pheromone trail and travels directly towards the female following the pheromone trail, but loses the trail prior to mate contact; (ii) contact events: the male crosses the pheromone trail to the point of mate contact, but fails to capture the female; and (iii) capture events: the male successfully tracked a female and captures her.

The swimming speed 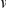 (mm s^−1^) over consecutive tracking intervals was estimated as 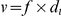, where *f* is the sampling rate of the camera (*f* = 25 frame s^−1^), and 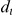 the distance (mm) between two points in a three-dimensional space. The distance 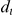 (mm) was computed from the 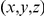 coordinates as 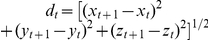, where 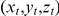 and 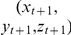 are the positions of a copepod at time 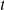 and 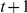, respectively. Average swimming speed and their standard deviations were measured over the duration of each individual track.

The complexity of swimming paths during non-mating and mating sequences was assessed using fractal analysis. In contrast to standard behavioral metrics such as turning angle and net-to-gross displacement ratio (NGDR), fractal analysis and the related fractal dimension 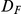 have the desirable properties to be independent of measurement scale and to be very sensitive to subtle behavioral changes that may be undetectable to other behavioral variables [16], [35], [36], [40], [41]. Fractal analysis has been applied to describe the complexity of zooplankton and ichtyoplankton swimming paths [33]–[36], [41]–[47]. The fractal dimensions of *T. longicornis* swimming paths were estimated using the box dimension method [41]. The box dimension method relies on the “*l* cover” of the object, i.e. the number of boxes of length *l* (or circles of radius *l*) required to cover the object. A more practical alternative is to superimpose a regular grid of boxes of length *l* on the object and count the number of ‘occupied’ boxes. This procedure is repeated using different values of *l*. The volume occupied by a swimming path is then estimated using a series of counting boxes spanning a range of volumes down to some small fraction of the entire volume. The number of occupied boxes increases with decreasing box size, leading to the following power-law relationship:
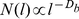
(2)where *l* is the box size, *N*(*l*) is the number of boxes occupied by the swimming path, and 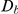 is the box fractal dimension. The fractal dimension 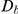 is estimated from the slope of the linear trend of the log-log plot of *N*(*l*) versus *l*; 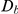 is bounded between 1 and 2 for linear swimming paths, and swimming paths so complex that they fill-in the two-dimensional space. To avoid potential biases related to both the anisotropy of the swimming paths and the initial orientation of the overlying three-dimensional grid of orthogonal boxes, for each box size *l* the grid was rotated in 5° increments from 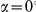 to 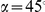 in the 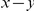 plane and from 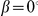 to 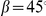 in the 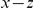 plane. The resulting distribution of fractal dimensions 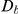 was averaged, and the resulting dimension 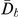 used to characterize the complexity of a swimming path.

The ability of males to detect female pheromone trails was quantified by the distance between the male and the female when the male initiates a tracking behavior, and the age of the female's trail upon detection by the male. The accuracy with which males followed females trails was estimated using the male-to-female displacement ratio, 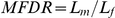, where 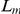 is the length of the male tracking trajectory and 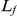 the length of the female trajectory [11]; *MFDR* = 1 when the male's trajectory is matching exactly the female's one, 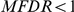 characterized males taking short-cuts along females's trajectories, and 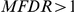 characterized males swimming with more frequent turns than females. The contact and capture rates were estimated as the number of contact and capture events normalized by the number of tracking events.

### Statistical analyses

Because the behavioral parameters considered in the present work were consistently non-normally distributed (*P*<0.01), pairwise comparisons were conducted using the Wilcoxon-Mann-Whitney *U*-test [48]. Multiple comparisons between groups of measurements were conducted using the Kruskal-Wallis test (KW test hereafter [49]), and a subsequent multiple comparison procedure based on the Tukey test was used to identify distinct groups of measurements [49]. Frequencies were compared using a χ^2^ test and the Fisher's exact test for frequencies with respectively more and less than 5 individuals [49]. All significativity levels were set at *P*<0.05.

## Results

Under conditions of both uncontaminated and contaminated seawater, a total of 439 swimming paths were observed for *T. longicornis* adult females, and 551 swimming paths for adult males, including 411 non-mating behavioral sequences and 140 mating behavioral sequences. This resulted in the analysis of 522,771 data points for adult females, and 631,136 and 73,907 data points for adult males non-mating and mating behavioral sequences, respectively (Table 1).

**Table 1:** Number of individual swimming paths (*N*) and related number of successive positions (*n*) obtained for *Temora longicornis* adult females and males under conditions of uncontaminated seawater (Control) and seawater contaminated at 0.01%, 0.1% and 1% with the water-soluble fraction of diesel oil.

	Control		0.01%		0.1%		1%	
	*N*	*n*	*N*	*n*	*N*	*n*	*N*	*n*
Females	107	120375	111	132301	120	149400	101	120695
Males								
non-mating	122	173850	102	157903	88	132765	99	166618
mating	41	25375	32	16123	28	13645	39	18764

### Seawater contamination and *Temora longicornis* swimming behavior

**Figure 2:**
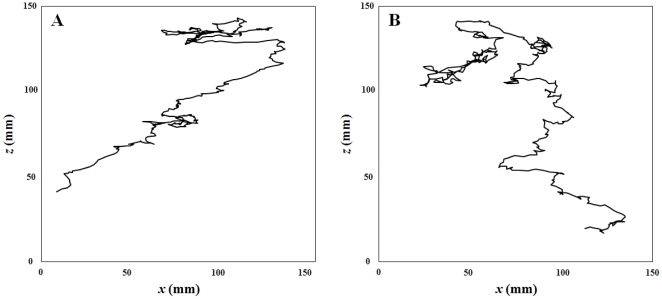
Example of two-dimensional swimming paths of *Temora longicornis* adult females (A), and adult males (B) during non-mating behavioural sequences.

**Figure 3:**
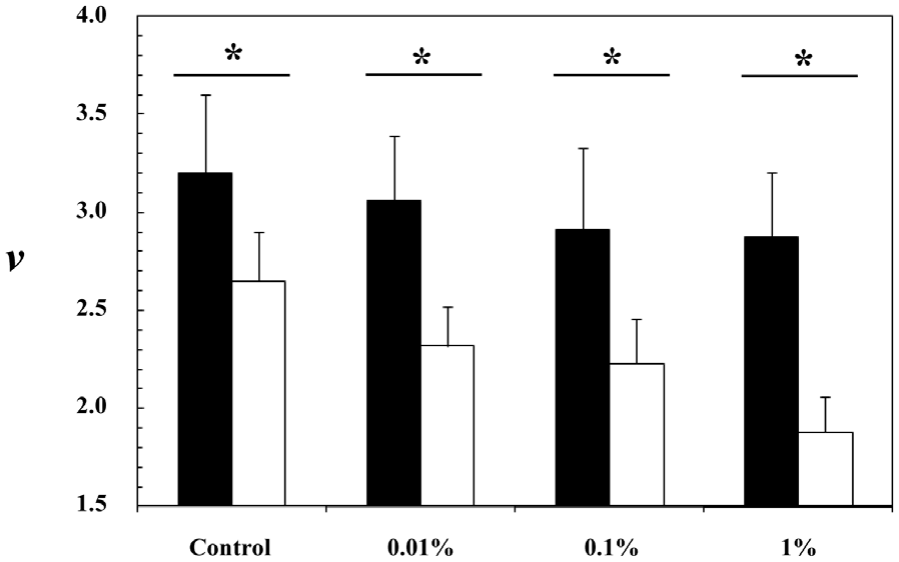
Swimming speed *v* (mm s^−1^) of *Temora longicornis* adult males during non-mating behavioural sequences (black bars) and adult females (white bars) in uncontaminated seawater (Control) and seawater contaminated at 0.01%, 0.1% and 1% with the water-soluble fraction of diesel oil. Error bars are standard deviations, and the symbol ‘*’ identifies significant differences (*P*<0.05) inferred for each experimental condition through pairwise comparisons using the Wilcoxon-Mann-Whitney *U*-test. Significant differences between treatments were conducted using the Kruskal-Wallis test, and a subsequent multiple comparison procedure based on the Tukey test.

Typical swimming trajectories of *T. longicornis* adult males and females are shown in Figure 2. Females consistently exhibited a cruising behavior, i.e. their rostro-caudal body axis was aligned with the direction of motion, whether they were swimming upward, downward or horizontally. In contrast to previous observations [11], [13], none of them were observed hovering (i.e. swimming upward at low speed, often with a horizontal component, with the rostro-caudal body axis oriented upward). During non-mating behavioral sequences, males swam significantly faster than females, in both uncontaminated and contaminated conditions (*P*<0.01; Fig. 3). The swimming speeds of females significantly differ between experimental conditions, and significantly decreased from uncontaminated to contaminated seawater (*P*<0.01; Fig. 3). In contrast, no significant differences were found in the swimming speeds of males between treatments (*P*>0.05; Fig. 3).

**Figure 4:**
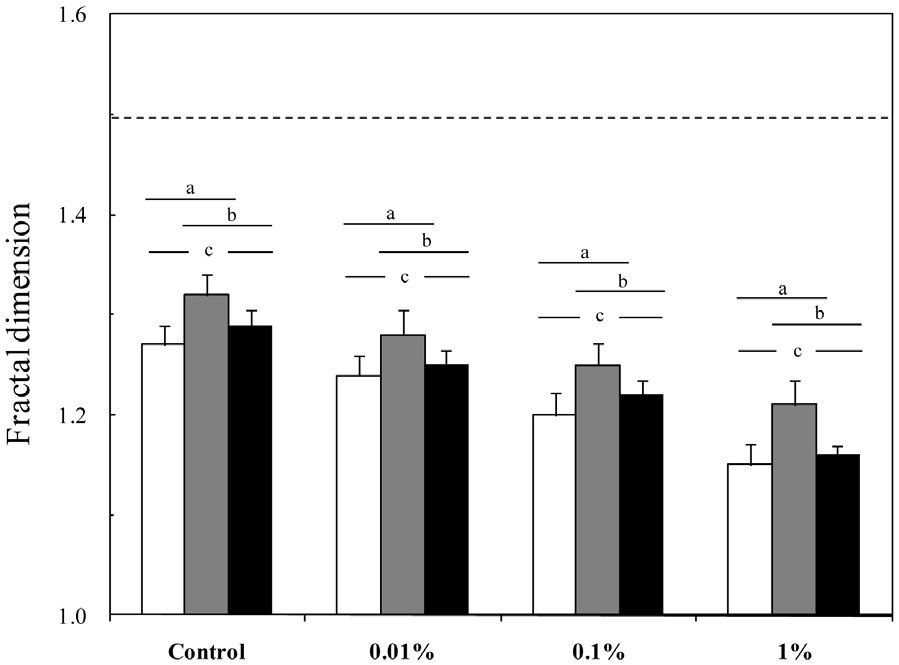
The box fractal dimension 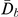
 **estimated from swimming paths of free swimming** ***Temora longicornis*** **adult females (white bars), and adult males during non-mating (grey bars) and mating behavioural (black bars) sequences in uncontaminated seawater and seawater contaminated at 0.01%, 0.1% and 1% with the water-soluble fraction of diesel oil.** The dashed line corresponds to the fractal dimension 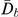 expected for a Brownian motion (i.e. normal diffusion). Error bars are standard deviations. The letters ‘a’, ‘b’ and ‘c’ identify significant differences (*P*<0.05) inferred for each experimental condition using the Kruskal-Wallis test, and a subsequent multiple comparison procedure based on the Tukey test.

The fractal dimensions 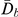 estimated for *T. longicornis* adult females and adult males during non-mating and mating behavioral sequences were significantly different for each experimental condition (*P*<0.05; Fig. 4). More specifically, 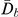 estimated for adult females and adult males during mating sequences did not significantly differ (*P*>0.05), and were both significantly smaller that those observed for adult males during non-mating sequences (*P*<0.05; Fig. 4). Finally, the fractal dimensions estimated for female swimming paths, and male swimming paths during non-mating and mating sequences significantly differ between treatments (*P*<0.05; Fig. 4). Specifically, 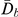 were consistently higher under conditions of WSF contaminations than in uncontaminated seawater, and significantly decreased with increasing WSF concentrations (*P*<0.05; Fig. 4).

### Seawater contamination and *Temora longicornis* mate-searching behavior

**Figure 5:**
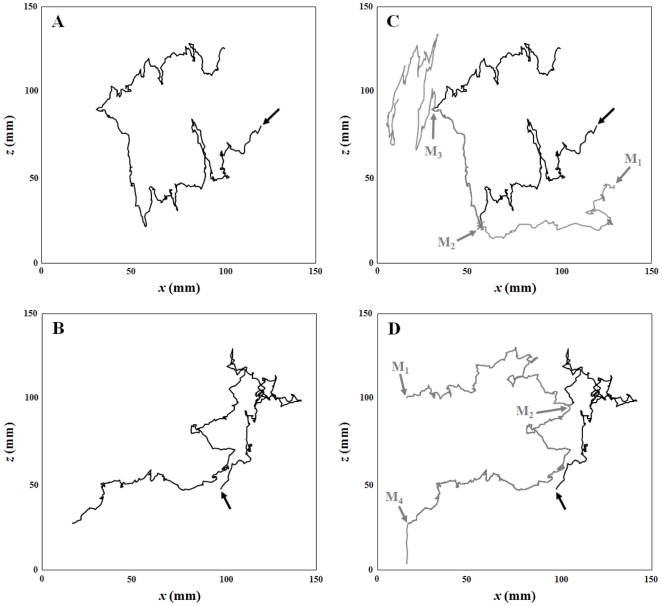
*Temora longicornis*. Two female swimming paths (A,B) that led to unsuccessful (C) and successful (D) male mate-searching behavioural sequences. The swimming paths of females and males are shown in black and grey, respectively. The black arrows indicate the beginning of the female swimming paths; the grey arrows indicate the beginning of male swimming paths (M_1_), the detection of the female pheromone trail by the male and the subsequent initiation of a mating behavioural sequence in which the male follows the female's trail (M_2_), a contact event where the male failed to capture the female (M_3_), and a contact event followed by a successful capture (M_4_) after which male and female sink slowly together.

**Figure 6:**
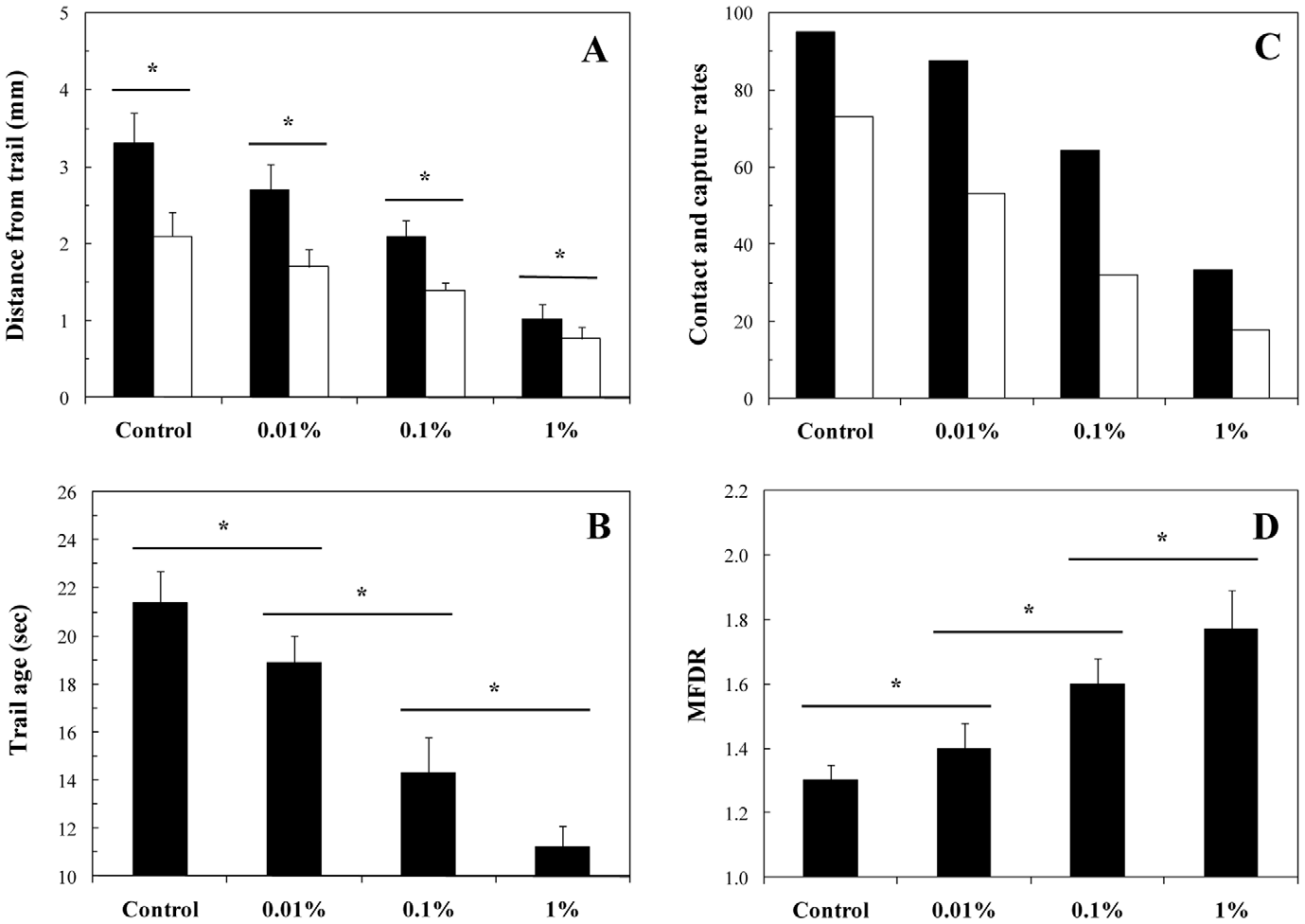
*Temora longicornis*. (A) Male distance from female track-line at detection of female trail (black bars) and during tracking (white bars). (B) Age of female's trail at detection by male. (C) Male-female contact rate (black bars) and capture rate (white bars). (D) Male-to-female displacement ratio (*MFDR*). Error bars are standard deviations. The symbol ‘*’ identifies significant differences (*P*<0.05) inferred for each experimental condition (A) through pairwise comparisons using the Wilcoxon-Mann-Whitney *U*-test, and between experimental conditions (B,D) through multiple comparisons conducted using the Kruskal-Wallis test, and a subsequent multiple comparison procedure based on the Tukey test used to identify distinct groups of measurements.

Figure 5 shows unsuccessful and successful mate-searching behavioral sequences; mate-searching proceeds through a trail-following strategy in which the females leave a discrete pheromone trail that mate-seeking males detect and follow until contact (Fig. 5a, b) and eventually capture (Fig. 5b) of the female. Males also exhibited the spinning behavior previously reported for *Temora longicornis* and *Temora stylifera* upon detection of the pheromone trail, and continued spinning throughout the pursuit of the female [11], [13]. In uncontaminated seawater, males detected pheromone trails within 3 to 4 mm (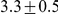 mm; 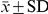) of the female track-line, and followed trails up to 29.9 sec old (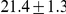 sec; Fig. 6a, b). In WSF contaminated seawater, the detection distance and the age of trail at detection significantly differ from those observed in control seawater, and significantly decrease with increasing WSF concentrations (Fig. 6a, b). Offset distances also significantly decreased with WSF concentrations (Fig. 6a). Female pheromone trails were followed at offset distances ranging from 1.2 to 3.1 mm (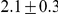 mm; Fig. 6a) from the female track line at an increasing velocity ranging from 8.5 to 24.1 mm s^−1^, which was significantly higher than the velocity observed during non-mating behavior (2.1 to 11.3 mm s^−1^).

In uncontaminated seawater males tracked pheromone trails in the incorrect direction in 4 of the 41 tracking events observed (9.8%), and all of them exhibited a ‘back-tracking’ behavior along trails in the correct direction. None of those males lost the female trails. Over the 41 tracking events, 39 (95.1%; Fig. 6c) led to a contact with a female (Fig. 5c) and 26 (63.4%; Fig. 6c) to a successful capture (Fig. 5d). During the 13 unsuccessful captures, 4 females escaped before the contact actually took place (suggesting that they hydromechanically sensed the male approach) and 9 escaped from the males after contact; it is, however, unclear if they were rejected by the males, or escaped from the male grasp.

In contrast, in contaminated seawater an increasing proportion of males followed female trails in the wrong direction (Table 2), and only 20.0 to 58.3% of them exhibited a back-tracking behavior, with this proportion significantly decreasing with increasing WSF concentrations (*P*<0.05; Table 2). None of the observed back-tracking behavior led to a successful contact with a female. The contact and capture rates significantly decreased with increasing WSF concentration (*P*<0.05; Fig. 6c). Nearly all the unsuccessful capture events resulted from females escaping before the contact with the male actually took place (Table 2). This is consistent with the increased stress levels demonstrated in *Centropages hamatus* adult females in response to naphthalene contamination [15], and may indicate an increase in the role played by mechanoreception when chemosensory abilities are hampered.

**Table 2:** Behavioural properties of adult males *Temora longicornis* during female tracking events observed in uncontaminated seawater (Control) and seawater contaminated at 0.01%, 0.1% and 1% with the water-soluble fraction of diesel oil.

	Control		0.01%		0.1%		1%	
	N	%	N	%	N	%	N	%
Tracking events	41		32		28		39	
Correct direction	37	90.2	20	62.5	16	57.1	19	48.7
Incorrect direction	4	9.8	12	37.5	12	42.9	20	51.3
Back-tracking	4	100.0	7	58.3	4	33.3	4	20.0
Contact	39	95.1	28	87.5	18	64.3	13	33.3
Successful capture	26	63.4	17	53.1	9	32.1	7	17.9
Unsuccessful capture	13	31.7	11	34.4	9	32.1	6	15.4
Female escape before contact	4	30.8	10	90.9	9	100.0	6	100.0
Female escape after contact	9	69.2	1	9.1	0	0.0	0	0.0

Frequencies were compared using a 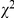 test and the Fisher's exact test for frequencies with respectively more and less than 5 individuals.

Finally, the accuracy with which males followed female trails was significantly smaller in uncontaminated seawater (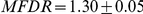) than in WSF contaminated seawater. Specifically, 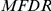 significantly increased with increasing WSF concentrations (Fig. 5d).

## Discussion

### Seawater contamination and *Temora longicornis* swimming behavior

Changes in motion behavior, as a response to stress induced by exposure to organic or inorganic pollutants, have been observed in a range of aquatic invertebrates such as *Artemia salina* [50], *Balanus amphitrite* [51]–[53], *Brachionus calyciflorus* [54], [55], *Daphnia magna* [56]–[62], *Hippolyte inermis* [63] and *Daphniopsis australis* [41], but still rarely on copepods [15], [16], [34], [41], [64].

In the present work, *Temora longicornis* adult males and females exhibited distinct behavioral responses to WSF contaminations. Specifically, the swimming speeds of females consistently decreased from uncontaminated to contaminated seawater, while no significant differences were detected between treatments in male swimming speeds (Fig. 3). This is consistent with the behavioral responses previously observed in zooplankton following water contamination; they range from hypoactivity to hyperactivity, depending on the species, concentration and nature of the contaminant, and exposure time. For instance, *Daphnia magna* decreased their swimming speed after a several day exposure to cadmium [57] and a 9-h exposure to copper at 30 µg l^−1^ [59]. In contrast, no changes were recorded following a 24-h exposure to copper at 5 µg l^−1^ [59], and an increase in swimming speed occurred after a 24-h exposure to methyl-paraoxon at 0.7 µg l^−1^ [62]. More specifically, the differences observed here between *T. longicornis* males and females further suggest that their chemosensory systems may have different sensitivity, females being more acute than males to the water-soluble fraction of diesel oil.

The decrease in female swimming speed and the steady male swimming speed with increasing WSF concentration contrasts with recent observations conducted on the estuarine calanoid copepod *Eurytemora affinis*, which consistently increased their swimming speed immediately after exposure to 2 µg l^−1^ of 4-nonylphenol and nonylphenol-ethoxy-acetic-acid [65]. However, because the swimming behavior of *E. affinis* was recorded before and after the injection of 15 µl of test solution, the behavioral observations conducted in contaminated water are more likely to result from the exposure to a gradient than a background concentration of nonyphenols. This is consistent with *T. longicornis* and *E. affinis* females escaping at high velocities when reaching patches of WSF contaminated seawater [16], hence with distinct behavioral reactions following an exposure to a background concentration of contaminants and a gradient of contaminants. This also generalizes to seawater contaminated by anthropogenic pollutants previous work showing the ability of copepods to detect and react to physical and chemical gradients naturally occurring in the ocean [66]–[68].

Swimming speed has been considered as a behavioral end-point for sub-lethal toxicity bioassays [53], [69]. However, it has also been shown that conventional metrics such as swimming speed were less sensitive than fractally-derived metrics to describe zooplankton behavioral properties in general [35], [36], [40] and under various conditions of stress in particular [34], [41]. For instance, the swimming speed of the calanoid copepod *Centropages hamatus* is not significantly different in seawater contaminated by naphthalene for concentrations ranging from 50 to 10,000 µg l^−1^ than in uncontaminated seawater [15]. In contrast, the cumulative distribution functions of move duration significantly differ between each naphthalene concentration, indicating an increase in behavioral stress with naphthalene concentrations [15]. The fractal dimension of the three-dimensional trajectories and the cumulative probability distribution functions of move lengths of five species of common calanoid copepods (*Acartia clausi*, *Centropages typicus*, *Paracalanus parvus*, *Pseudocalanus elongatus* and *Temora longicornis*) were also more sensitive to experimental stress than swimming velocity [34]. Similar conclusions were reached from the assessment of salinity stress in *Daphniopsis australis* and temperature stress in *Temora longicornis* [41]. These results are consistent with the significant decrease in the fractal dimensions of male three-dimensional trajectories with increasing WSF concentrations (Fig. 4), indicating a decrease in the complexity of swimming patterns under conditions of WSF contamination. This is also congruent with the generally reported decrease in behavioral complexity under stressful conditions for both invertebrates [15], [34], [41] and vertebrates [70]–[75], and specifically for organisms contaminated by pollutants such as tetrachloroethylene [76] and lead [77].

### Seawater contamination and *Temora longicornis* mate-searching behavior

The behavioral observations conducted here on *T. longicornis* in uncontaminated seawater and WSF contaminated seawater are consistent with mate-searching events previously reported for this species; see e.g. [11], [13], [37]–[39]. This suggests that the contamination of seawater by the water-soluble fraction of diesel oil did not modify *T. longicornis* overall mate-searching strategies. However, the significant decrease with increasing WSF concentrations in the male distance from female track-line at detection of female trail (Fig. 6a) and during tracking (Fig. 6b) as well as the increase in male-to-female displacement ratio with increasing WSF concentration (Fig. 6d) indicate that the chemosensory ability of male *Temora longicornis* to successfully detect and track females is affected by the water-soluble fraction of diesel oil for concentrations as low as 0.01%. This ultimately leads to 2.8- and 4.1-fold decreases in mate encounter and capture rates, respectively (Fig. 6c, Table 2).

The observed changes in the swimming behavior of male *T. longicornis* may be directly related to changes in the ability of males to detect the females pheromone trail, which in turn may be the result of two non-conflicting mechanisms: the water-soluble fraction of diesel oil directly disturbs the males' chemosensory system, and/or a chemical interaction of the water-soluble fraction of diesel oil with the pheromone molecules changes the molecule structure, hence renders it unrecognizable by the males chemosensory system. A decrease in the efficiency of male sensory system is consistent with the anomalous neurotransmission resulting from acetylcholine esterase inhibition following exposure to pollutants [78]; see also [62] and references therein.

The behavioral changes observed in male swimming patterns may also be a consequence of a decrease in the rate of pheromone production by the females. In response to stress and to the related reduction in the energy balance, organisms typically perform a series of compensatory responses to improve the probability of survival, involving the endocrine system and behavior [72], [79]–[81]. It is hence reasonable to think that *T. longicornis* females may control the probability of mating by reducing, and ultimately stopping, the production of pheromones under stressful conditions such as contamination by the soluble-fraction of diesel oil. This hypothesis is consistent with previous evidence of reduced egg production in copepods in seawater contaminated by polycyclic aromatic hydrocarbons [7]–[9]. The changes observed in female swimming behavior (Fig. 3) are also congruent with an adaptive strategy leading to decrease mate encounter rates. The encounter rate 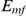 between a male and a female swimming respectively at velocities *u* and *v* is given by [82]:
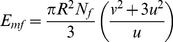
(3)when 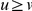. 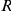 is the perceptive distance of the male, and 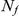 the female density. It is straightforward from Eq. (3) that the decreasing swimming speed of females and the steady swimming speed of males under increasing WSF concentrations (Fig. 3) leads to a decrease in mate encounter rates. Similarly, a modeling approach demonstrated that the number of encounters *E_mf_* is a linear function of the three-dimensional fractal dimensions defined as 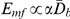, where the pre-factor 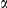 depends on the motion behavior 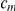 and female density 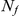 as 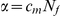 [83]. The decreasing fractal dimensions observed for both males and females under conditions of WSF water contamination (Fig. 4) hence also leads to decrease mate encounter rates.

### Seawater contamination and zooplankton: ecological implications

The changes observed in the swimming behavior of both males and females *T. longicornis* in response to seawater contaminated by the water-soluble fraction of diesel oil have far reaching implications for copepod ecology as in some species of planktonic copepods population growth rate is heavily constrained by relatively low male mating capacity and abundance, and the related fertilisation limitation [84]. In this context, a decrease in encounter and capture rates due to hydrocarbon contamination may profoundly impact the structure and function of the pelagic ecosystems through a destabilization of copepod population dynamics. The ability of copepods to detect, avoid and escape localized patches contaminated by the soluble fraction of diesel oil [16] may limit the behavioral effect reported here for point source contaminations. However, my results suggest that even low background concentrations of hydrocarbons may have more pernicious effects on population dynamics than the previously reported sub-lethal effects on copepod physiology, feeding and fecundity [7]–[9]. These findings prompt critical considerations of behavioral adaptations in copepods (and potentially other invertebrates) and the incorporation of behavioral changes induced by hydrocarbon contamination into general predictive models of planktonic copepod population dynamics based on the role of individual swimming behavior [85], [86].
